# Hemodynamic Changes During Heart Displacement in Aorta No-Touch Off-Pump Coronary Artery Bypass Surgery: A Pilot Study

**DOI:** 10.21470/1678-9741-2018-0090

**Published:** 2018

**Authors:** Alexandre R. Carvalho, Solange Guizilini, Gustavo M. Murai, Isis Begot, Isadora S. Rocco, Nelson A. Hossne Jr, Eduardo G. Chamlian, João Manoel T. Santos, Ricardo A. Macedo, Gustavo C.O. Lisboa, Alberto C. Nasciutti, Carlos Eduardo R. Santos, João Paulo M. Figueiredo, Walter J. Gomes

**Affiliations:** 1 Policlin Hospital, São José dos Campos, SP, Brazil.; 2 Pirajussara General Hospital/SPDM and Clinics Hospital Luzia de Pinho Melo/SPDM, São Paulo, SP, Brazil.; 3 Discipline of Cardiovascular Surgery, Escola Paulista de Medicina and São Paulo Hospital – Universidade Federal de São Paulo (Unifesp), São Paulo, SP, Brazil.

**Keywords:** Coronary Artery Bypass, Coronary Artery Bypass, Off-Pump, Stroke, Intracranial Embolism, Hemodynamics

## Abstract

**Objective:**

To evaluate the sequential changes of hemodynamic and metabolic parameters in patients who underwent aorta no-touch off-pump coronary artery bypass surgery (OPCAB).

**Methods:**

Prospective study involving twenty-seven consecutive patients who underwent aorta no-touch OPCAB. The FloTrac/PreSep/Vigileo™ system (Edwards Lifesciences) was used to continuously record heart rate (HR), mean arterial blood pressure (MABP), central venous pressure (CVP), continuous cardiac index (FCI), stroke volume (SV), stroke volume variation (SVV), and central venous oxygen saturation (ScvO_2_). The parameters were assessed 5 min before, during and 5 min after each anastomosis (left anterior descending [LAD], posterior descending [PD], obtuse marginal [OM] and diagonal [Dg]). Postoperative lactate was also evaluated.

**Results:**

There was no significant change in HR and MABP for all anastomoses, except for MABP during PD grafting (-10.1±2.7 mmHg, *P*=0.03). There was a significant decrease in ScvO_2_ only during PD and OM anastomoses (-9.4±0.4, *P*=0.03; -4.4±0.4, *P*=0.02; respectively). CVP drop after PD manipulation was strongly associated with a higher lactate during the first hours after surgery (r=-0.82; *P*=0.001). These hemodynamic changes were transient and entirely recovered after the heart was returned to its anatomical position. No significant differences were observed in FCI, SVV, or the systemic vascular resistance index (SVRI) during all anastomoses, except for a drop in SVRI during PD grafting (-8.03±2.3, *P*=0.007). SV tended to decrease during the procedure in all territories, but with statistically significant drop only in PD and OM grafting (-10.4±1.2, *P*=0.02; -13.6±5.1, *P*=0.007; respectively).

**Conclusion:**

Heart displacement for performing aorta no-touch OPCAB is well tolerated, with transient and endurable hemodynamic variations.

**Table:** 

Abbreviations, acronyms & symbols				
CABG	= Coronary artery bypass grafting		LVEF	= Left ventricular ejection fraction
CCO	= Continuous cardiac output		MABP	= Mean arterial blood pressure
CO	= Cardiac output		OM	= Obtuse marginal
CVP	= Central venous pressure		OPCAB	= Off-pump coronary artery bypass surgery
Dg	= Diagonal		PCI	= Percutaneous coronary intervention
ECG	= Electrocardiogram		PD	= Posterior descending
FCI	= Continuous cardiac index		RITA	= Right internal thoracic artery
HR	= Heart rate		ScvO_2_	= Central venous oxygen saturation
ITAs	= Internal thoracic arteries		SV	= Stroke volume
LAD	= Left anterior descending		SVRI	= Systemic vascular resistance index
LITA	= Left internal thoracic artery		SVV	= Stroke volume variation
LV	= Left ventricle			

## INTRODUCTION

The aorta no-touch off-pump coronary artery bypass surgery (OPCAB) has been the recommended technique for treatment of patients with high risk for neurological damage or stroke^[1]^. The concept stems from avoiding any ascending aorta manipulation, virtually eliminating the risk of embolism of aortic wall debris into the brain circulation, utilizing the off-pump beating heart surgery technique^[2]^. However, the displacement of the heart to achieve suitable exposure for graft construction elicits hemodynamic changes, potentially requiring conversion to on-pump surgery and increasing morbimortality risk^[3]^. Early detection of premonitory hemodynamic alterations leading to conversion may help avert this dreadful complication.

The FloTrac/PreSep/Vigileo™ system (Edwards Lifesciences, Irvine, CA, USA) is a less invasive monitoring device allowing continuous determination of cardiac output (CO) and other hemodynamic variables using pulse wave analysis, coupled with mixed central venous oxygen saturation (ScvO_2_) assessment^[4]^.

Therefore, the aim of this study was to evaluate the sequential changes of hemodynamic variables during coronary artery anastomoses in patients who underwent aorta no-touch OPCAB using the FloTrac/PreSep/Vigileo™ system.

## METHODS

This prospective study enrolled twenty-seven consecutive patients (16 males, 11 females; mean age, 63.7±9.6 years) who underwent elective first-time aorta no-touch OPCAB. After the Institutional Ethical Committee's approval, informed consent was obtained from all the patients. All operations were performed by a single surgeon (WJG) to eliminate technical variability.

In the operating room, electrocardiogram (ECG) leads were monitored, the radial artery was cannulated for continuous monitoring of arterial blood pressure and blood gas analysis and connected to a FloTrac™ pressure transducer. The PreSep™ central venous oximetry catheter was inserted through the right jugular vein, the Vigileo™ system was next loaded with the patient's demographic data, pressure was zeroed and set to display variables' measurement within the past 60 seconds.

Anesthetic technique was standardized for all patients. Anesthesia was induced with 2.0–3.0 mg of midazolam, 1.0–3.0 mg/kg of sufentanil, and 50 mg of rocuronium and it was maintained with 0.2–0.5 vol% of isoﬂurane and continuous intravenous infusion of 0.5–1.5 mg/kg/min of sufentanil and vecuronium. Ventilation was controlled with oxygen-air mixture (FiO_2_ 0.6) to maintain end-tidal CO_2_ in 35–38 mmHg. For preventing hypothermia, the operating room's temperature was maintained above 24˚C and the patients were continuously rewarmed with warm mattress. After median sternotomy, the internal thoracic arteries (ITAs) were taken-down from the inner chest wall in skeletonized fashion and heparin was given to attain activated clotting time > 250 seg. After pericardial longitudinal opening, the heart was inspected, and pericardial stay sutures were placed, deeper in the left side than in the right. Additionally, one or two deep pericardial stitches were placed close to the left inferior pulmonary vein. All these sutures together, once forcefully pulled up, improved the exposure of the lateral and inferior aspects of the heart. The application of a suction-type tissue stabilizer (Octopus Tissue Stabilizer System, Medtronic Inc., Minneapolis, MN, USA) completed the task of a suitable exposure of the coronary arteries to be grafted. The left anterior descending (LAD) is the first coronary artery in the grafting sequence, followed by the posterior descending (PD), obtuse marginal (OM), and diagonal (Dg) arteries. No distal coronary snaring, CO_2_ blower, or intra-coronary shunts were used in this series, in accordance with our usual OPCAB practice.

Hemodynamic stability was maintained, once necessary, with transitory use of inotropes (dobutamine infusion), vasopressors (noradrenaline), and volume expansion with crystalloid solutions. During graft anastomosis to OM and PD, patients were placed in the Trendelenburg position, and the table was rotated slightly to the right.

**Fig 1:**
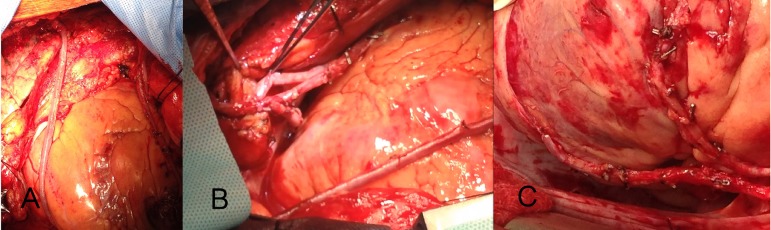
*A*. The pericardium is not split over the aorta. Bilateral ITA grafts (LITA-LAD and RITA-OM in Y fashion) and vein graft to PDA anastomosed end-to-end with the RITA proximal stump). *B*. RITA and saphenous vein grafts anastomosed to LITA, in addition to RITA-vein-PDA. *C*. LITA sequential grafting to LAD and Dg and RITA free graft to OM.

The OM was always revascularized using right internal thoracic artery (RITA) grafts (16/27), either *in situ* routed through the transverse sinus or as a free graft attached end-to-side to the left internal thoracic artery (LITA). The vein graft to PD was connected to the RITA's proximal stump (17/27). Figure 1.

All proximal grafts were attached to the ITAs, and the ascending aorta remained untouched throughout all the operations; most of the times, the pericardium over the aorta was not split to completely avert any aorta manipulation.

Protamine was administered to fully reverse the heparin effect after the completion of revascularization.

The FloTrac/PreSep/Vigileo system™ (Edwards Lifesciences) was utilized to continuously record hemodynamic variables: heart rate (HR), mean arterial blood pressure (MABP), central venous pressure (CVP), continuous cardiac index (FCI), stroke volume (SV), stroke volume variation (SVV), and ScvO_2_. The systemic vascular resistance index (SVRI) derived from collected parameters was calculated. The FloTrac™ system utilizes the radial artery line for analysis of the arterial waveform to calculate CO and other parameters derived from CO data. The PreSep™ central venous oximetry catheter provides a central venous line for volume administration and once connected to the Vigileo™ monitor, it allows continuous measurement of CVP and ScvO_2_.

The hemodynamic variables were assessed 5 minutes before the heart's displacement for anastomosis, during graft construction, and 5 minutes after each anastomosis (LAD, PD, OM, and Dg). The last measurement was performed with the heart returned to its anatomical position. Postoperative lactate was also evaluated.

### Statistical Analysis

Categorical data were presented in absolute (n) and relative (%) frequencies. All hemodynamic data were expressed as the mean value ± standard deviation. The Shapiro-Wilk test was applied to tested variables for normality distribution. Hemodynamic changes during the heart's displacement for each coronary anastomosis were compared using ANOVA for repeated measures. The drop in hemodynamic variables was presented by the difference between the initial or after the procedure value minus the during the heart's displacement value. A statistically significant level was considered when *P*-value<0.05. Statistical analyzes were carried out by GraphPrism 7.0 Software (GraphPad, USA).

## RESULTS

All patients were successfully operated on according to the scheduled protocol. Demographic data are listed in Table 1. No patient presented signs of intraoperative myocardial infarction, as assessed by abnormal elevation of serial cardiac enzymes or 12-lead ECG changes.

**Table 1:** Demographic, clinical, and operative data of participants.

Variables	n = 27
Age (years)	63.7±9.6
Male/Female (n)	16-11
Body mass index (kg/m^2^)	26.6±4.5
Hypertension, n (%)	22 (81.48)
Diabetes, n (%)	12 (44.44)
Current smokers, n (%)	6 (22.22)
Previous MI, n (%)	10 (37.03)
LVEF	0.61±0.10
β-blockers, n (%)	25 (92.59)
Nitrate, n (%)	11 (40.74)
Calcium channels blockers, n(%)	6 (22.22)
ACE inhibitors, n (%)	12 (4.44)

Data are presented in mean ± standard deviation.

ACE=angiotensin converting enzyme; β=blockers medication doses; LVEF=left ventricular ejection fraction; MI=myocardial infarction

**Fig. 2:**
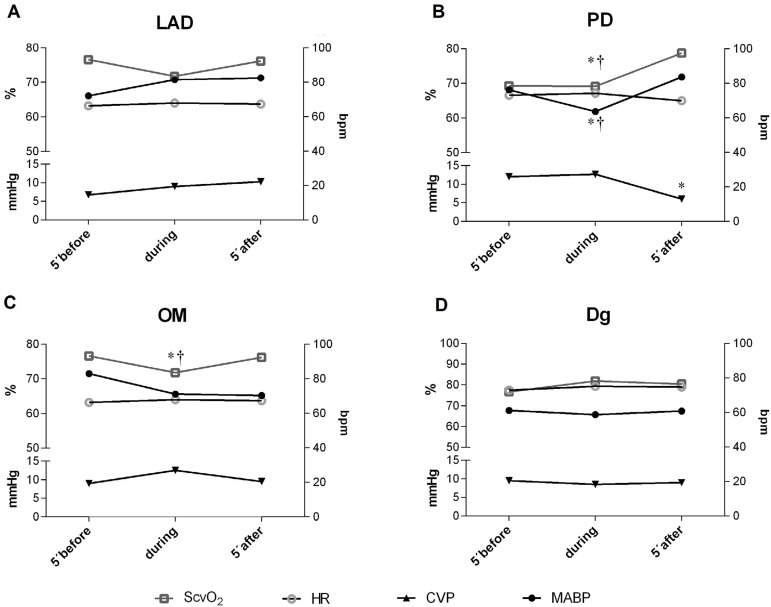
Hemodynamic changes during coronary artery bypass graft anastomosis. CVP=central venous pressure; Dg=diagonal; HR=heart rate; LAD=left anterior descending; MABP=mean arterial blood pressure; OM=obtuse marginal; PD=posterior descending; ScvO_2_=central venous oxygen saturation

The mean number of anastomoses was 2.7 per patient and the left ventricular ejection fraction (LVEF) varied between 0.31 and 0.71 (mean 0.61±0.10); four patients had LVEF below 0.45. There was no significant change in HR and MABP for all anastomoses, except for MABP during PD grafting (-10.1±2.7 mmHg, *P*=0.03; Figure 2). The effects on ScvO_2_ were variable, but no change above 10% was observed. There was a significant decrease in ScvO_2_ only during PD and OM anastomoses (-9.4±0.4, *P*=0.03; -4.4±0.4, *P*=0.02; respectively, Figure 2). CVP drop after PD manipulation was strongly associated with a higher lactate during the first hours after surgery (r=-0.82; *P*=0.001). These hemodynamic changes were transient and entirely recovered after the heart was returned to its anatomical position.

**Fig. 3:**
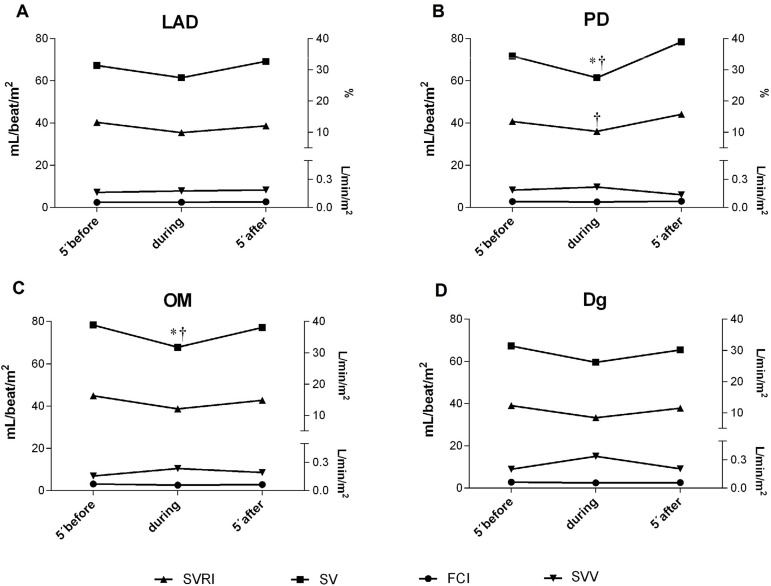
Hemodynamic responses during coronary artery bypass graft anastomosis. Dg=diagonal; FCI=continuous cardiac index; LAD=left anterior descending; OM=Obtuse marginal; PD=posterior descending; SV=stroke volume; SVRI=systemic vascular resistance index; SVV=stroke volume variation

No significant differences were observed in FCI, SVV, or SVRI during all anastomoses, except for a drop in SVRI during PD grafting (-8.03±2.3, *P*=0.007; Figure 3). SV tended to decrease during the procedure in all territories, but with statistically significant drop only in PD and OM grafting (-10.4±1.2, *P*=0.02; -13.6±5.1, *P*=0.007; respectively, Figure 3).

## DISCUSSION

The main findings of this present study revealed that hemodynamic alterations during distal graft construction in aorta no-touch OPCAB were more commonly related to PD and OM anastomoses (inferior and lateral walls, respectively), but these were transient and well tolerated, with the heart fully recovering when it was returned to its anatomical place. The heart displacement for LAD anastomosis is the least affected by hemodynamic changes, that is why it is performed first in the sequence. Moreover, early revascularization of the left ventricle (LV) anterior wall affords protection to the bulk of the LV myocardium, therefore allowing for safer heart displacement to accomplish the operation.

The hemodynamic compromise is an important concern with this technique, caused by displacement of the beating heart, which is vital for optimal exposure of the circumﬂex and PD artery. However, innovative improvements for coronary artery visualization^[5,6]^ and advanced methods for heart positioning and anastomotic site stabilization^[7,8]^ improved safety and technical quality, resulting in wider acceptance and extended use of this operation.

Coronary artery bypass grafting (CABG) has consistently been demonstrated to be the most effective therapy for the treatment of patients with advanced obstructive atherosclerotic coronary artery disease, providing reduction of the long-term risk of death and myocardial infarction, compared to percutaneous coronary intervention (PCI)^[1]^. However, the increased risk of stroke following CABG remains a major drawback for surgical revascularization compared with PCI, which is mostly related to the aorta manipulation^[9]^.

Several meta-analyses revealed that OPCAB significantly reduces the incidence of postoperative neurological complications compared to on-pump CABG^[10,11]^, although an increased risk still persists owing to the need of ascending aorta clamping to construct the proximal anastomosis^[12]^.

More than 60 randomized trials so far have compared OPCAB with on-pump CABG, allowing for construction of several meta-analyses of these trials, all coming to a similar conclusion: OPCAB significantly reduced short-term rates of stroke and renal failure but did not reduce the risk of mortality or myocardial infarction in low and mixed risk patients. Specific studies targeting high-risk patients found a significant reduction in mortality with OPCAB compared with on-pump CABG, although with higher rates of repeat revascularization^[13]^.

In the CORONARY trial^[14]^, the largest prospective randomized trial performed so far comparing on- and off-pump surgery, the rate of the composite outcome of death, stroke, myocardial infarction, renal failure, or repeat revascularization at 5-years follow-up was similar among patients who underwent OPCAB and on-pump CABG.

Recent network meta-analysis demonstrated that avoidance of aortic manipulation in OPCAB may decrease the risk of post-operative stroke, especially in higher-risk patients. Additionally, the study pointed out that the elimination of cardiopulmonary bypass may reduce the risk of short-term mortality, renal failure, atrial fibrillation, bleeding, and length of intensive care unit stay^[15]^. Therefore, the aorta no-touch OPCAB means a step forward and a refinement of the outcomes provided by the earliest OPCAB technique.

In another seminal study, the no-aortic touch technique afforded the lowest risk for postoperative stroke in patients undergoing CABG. Clamping the aorta during CABG increased the risk of postoperative stroke, regardless of the severity of aortic disease^[16]^. An additional strategy to overcome this pitfall, the technique for anastomosis of the top end of the vein graft to the proximal stump of RITA in the anaortic (aorta no-touch) OPCAB, has been developed and extensively employed in this case series^[17]^.

In Brazil, off-pump surgery has been demonstrated to reduce short-term costs of the procedure in 25% compared to the on-pump CABG, as well as it is cost-effective in the 5-years follow-up comparative analysis of the MASS-III trial. In our country, with severe health budget constraints, this savings could increase the ability to care for patients by one quarter^[18-20]^.

OPCAB surgery has been questioned regarding its efficacy and safety in comparison to the conventional technique, especially with the outcomes related to incomplete revascularization and quality of grafts. However, the quality of anastomosis in off-pump surgery is directly related to the surgeon's experience. While experienced surgeons performing the technique reported the same degree of patency of the techniques over 8-years follow-up^[21,22]^, in trials including less experienced surgeons, the OPCAB short- and long-term outcomes were inferior and led to high intraoperative conversion rates, as reported in the ROOBY trial^[23]^. Similarly, experienced surgeons tend to achieve higher rates of complete revascularization in the OPCAB technique.

Based in these recent evidences, the aorta no-touch OPCAB has been the recommended technique for patients with cerebrovascular disease and/or calciﬁcation or atheromatous plaque in the ascending aorta. Furthermore, it should be favored in patients with high-risk factors such as old age, left ventricular impairment, and renal failure.

Worsening of cardiac function due to heart's dislodgement during OPCAB can result in obstruction of the right ventricular outflow tract and failure in LV pumping mechanism. These changes elicit reduced CO, hypotension, and myocardial ischemia, potentially leading to cardiac arrest and urgent need to on-pump conversion. Then, likely benefits could be provided by real-time less invasive monitoring system allowing continuous determination of CO and other hemodynamic variables in OPCAB, and letting promptly correction or earlier conversion to on-pump technique^[24]^. Postoperative complications and mortality are markedly higher in patients with hemodynamic instability who underwent a delayed switch to an on-pump CABG^[24]^.

The number of coronary artery anastomoses during OPCAB is increasing, more arterial grafts are being performed, and then the requirement of proper and accurate management of hemodynamic and metabolic parameters during coronary anastomosis emerges. Apart from intravenous ﬂuid loading and head-down (Trendelenburg) position to compensate the drop of mean arterial pressure and CO, the use of a combination of inotropic drugs should be more intensively evaluated in this setting. Particularly, ScvO_2_ reﬂects hemodynamic changes earlier compared to the CO measured every 60 seconds and it has critical signiﬁcance since it represents both delivery and consumption of oxygen. Although measurement of the CO level is too slow for monitoring the cardiac status during the manipulation and tilting of the heart, the CO is still useful as a trend monitor during the entire surgical period. Ultimately, the hemodynamic changes during OPCAB are also heavily dependent on the surgical technique, surgeon's skill, and the operative team's experience.

In recent years, the FloTrac/Presep/Vigileo™ has been introduced into clinical practice, establishing a new concept of a semi-invasive device based on arterial waveform analysis for FCI evaluation and ScvO_2_ monitoring, along with patient's anthropometric data, skipping the necessity of calibration by thermodilution. Broch et al.^[25]^ comparatively evaluated the accuracy of FCI determination based on arterial waveform analysis with transpulmonary thermodilution in patients scheduled for elective CABG, showing a moderate, but signiﬁcant correlation between pulse contour FCI and thermodilution FCI, both before and after cardiopulmonary bypass. Not only the uncalibrated semi-invasive monitoring system was capable to reliably measure FCI compared with transpulmonary thermodilution in patients undergoing elective CABG, but additionally the semi-invasive monitoring device was able to detect hemodynamic changes and trends.

Yet, Jo et al.^[26]^ compared the FCI measured by the FloTrac/Vigileo™ system to that obtained by a pulmonary artery catheter in patients with decreased LVEF and low CO status during OPCAB. The FCI was reliable at all points of measurement and the authors concluded that the CO measured by the FloTrac/Vigileo™ system was consistent in patients with decreased LVEF and low CO status during OPCAB, even during the period of heart's displacement and OM grafting^[26]^. Conversely, other studies reported lower accuracy of uncalibrated arterial waveform analysis of SVV to predict ﬂuid responsiveness in patients with impaired LVEF and low CO, especially those with FCI < 2.2 L/min/m^2^^[27,28]^.

The routine use of continuous cardiac output (CCO) and ScvO_2_ monitoring for OPCAB is still a matter of debate, related to the question of whether increasing the degree and complexity of invasive monitoring will lead to improved outcomes. These devices may provide valuable information from a trending viewpoint, which may be useful for hemodynamic management in intra- and postoperative period. Accurate and timely data regarding these endpoints may signify the difference between carry-on the case off-pump or urgently convert it to on-pump surgery^[29]^. In this case series, no premonitory changes that could trigger an alarm for shifting the operative strategy were detected and that might be related to the number of patients included, so further increasing the caseload may be of help in elucidating this question.

## CONCLUSION

The heart displacement for performing aorta no-touch OPCAB in all main coronary arteries is well tolerated, with transient and endurable hemodynamic variations and no substantial influence on metabolic parameters or adverse clinical events. Close monitoring using the FloTrac/PreSep/Vigileo™ system may be valuable for prompt identification of significant hemodynamic and metabolic changes and implementation of proper intervention at the time of coronary artery anastomosis in patients undergoing aorta no-touch OPCAB.

**Table:** 

Authors' roles & responsibilities	
ARC	Substantial contributions to the conception or design of the work; or the acquisition, analysis, or interpretation of data for the work; final approval of the version to be published
SG	Substantial contributions to the conception or design of the work; or the acquisition, analysis, or interpretation of data for the work; final approval of the version to be published
GMM	Final approval of the version to be published
IB	Drafting the work or revising it critically for important intellectual content; final approval of the version to be published
ISR	Drafting the work or revising it critically for important intellectual content; final approval of the version to be published
NAHJ	Final approval of the version to be published
EGC	Drafting the work or revising it critically for important intellectual content; final approval of the version to be published
JMTS	Drafting the work or revising it critically for important intellectual content; final approval of the version to be published
RAM	Drafting the work or revising it critically for important intellectual content; final approval of the version to be published
GCOL	Final approval of the version to be published
ACN	Final approval of the version to be published
CERS	Final approval of the version to be published
JPMF	Final approval of the version to be published
WJG	Substantial contributions to the conception or design of the work; or the acquisition, analysis, or interpretation of data for the work; final approval of the version to be published
